# Specific Mutations in the Cholesterol-Binding Site of APP Alter Its Processing and Favor the Production of Shorter, Less Toxic Aβ Peptides

**DOI:** 10.1007/s12035-022-03025-9

**Published:** 2022-09-09

**Authors:** Linda Hanbouch, Béatrice Schaack, Amal Kasri, Gaëlle Fontaine, Eleni Gkanatsiou, Gunnar Brinkmalm, Elena Camporesi, Erik Portelius, Kaj Blennow, Gilles Mourier, Nicolas Gilles, Mark J. Millan, Catherine Marquer, Henrik Zetterberg, Lydie Boussicault, Marie-Claude Potier

**Affiliations:** 1 Paris Brain Institute, ICM, CNRS UMR7225-INSERM U1127-Sorbonne University Hôpital de La Pitié-Salpêtrière, 47 Bd de l’Hôpital, 75013 Paris, France; 2 Univ. Grenoble Alpes, CNRS, INP, TheRex Team, TIMC-IMAG, 38700 La Tronche, France; 3 Univ. Grenoble Alpes, CEA, CNRS, IBS, 38044 Grenoble, France; 4 Department of Psychiatry and Neurochemistry, Institute of Neuroscience & Physiology, the Sahlgrenska Academy at the University of Gothenburg, Mölndal, S-431 80 Sweden; 5 Clinical Neurochemistry Laboratory, Sahlgrenska University Hospital, S-431 80 Mölndal, Sweden; 6 Département Médicaments Et Technologies Pour La Santé (DMTS), Université Paris Saclay, CEA, INRAE, SIMoS, 91191 Gif-sur-Yvette, France; 7 Neuroscience Inflammation Thérapeutic Area, IDR Servier, 125 Chemin de Ronde, 78290 Croissy-sur-Seine, France; 8 Institute of Neuroscience and Psychology, College of Medicine, Vet and Life Sciences, Glasgow University, 62 Hillhead Street, Glasgow, G12 8QB Scotland; 9 Department of Neurodegenerative Disease, UCL Institute of Neurology, London, WC1N 3BG UK; 10 UK Dementia Research Institute at UCL, London, WC1E 6BT UK

**Keywords:** Alzheimer’s disease, Cholesterol, Amyloid precursor protein, Aβ, Mutant

## Abstract

Excess brain cholesterol is strongly implicated in the pathogenesis of Alzheimer’s disease (AD). Here we evaluated how the presence of a cholesterol-binding site (CBS) in the transmembrane and juxtamembrane regions of the amyloid precursor protein (APP) regulates its processing. We generated nine point mutations in the *APP* gene, changing the charge and/or hydrophobicity of the amino-acids which were previously shown as part of the CBS. Most mutations triggered a reduction of amyloid-β peptides Aβ40 and Aβ42 secretion from transiently transfected HEK293T cells. Only the mutations at position 28 of Aβ in the APP sequence resulted in a concomitant significant increase in the production of shorter Aβ peptides. Mass spectrometry (MS) confirmed the predominance of Aβx-33 and Aβx-34 with the APP^K28A^ mutant. The enzymatic activity of α-, β-, and γ-secretases remained unchanged in cells expressing all mutants. Similarly, subcellular localization of the mutants in early endosomes did not differ from the APP^WT^ protein. A transient increase of plasma membrane cholesterol enhanced the production of Aβ40 and Aβ42 by APP^WT^, an effect absent in APP^K28A^ mutant. Finally, WT but not CBS mutant Aβ derived peptides bound to cholesterol-rich exosomes. Collectively, the present data revealed a major role of juxtamembrane amino acids of the APP CBS in modulating the production of toxic Aβ species. More generally, they underpin the role of cholesterol in the pathophysiology of AD.

**Supplementary Information:**

The online version contains supplementary material available at 10.1007/s12035-022-03025-9.

## Introduction

Alzheimer’s disease (AD) is the most common form of dementia in the elderly population and is characterized by two prominent pathologies, extracellular amyloid-β (Aβ) containing plaques and intraneuronal fibrillary tangles comprised of aberrantly hyperphosphorylated tau protein [1]. Aβ peptides of various lengths are produced by sequential proteolysis of the transmembrane amyloid precursor protein (APP) by the β-secretase BACE1 and the γ-secretase both of which operate in the membrane bilayer [2]. Amyloidogenic APP processing predominantly occurs in the endolysosomal compartment following clathrin-dependent APP internalization [3].

There is considerable interest in endogenous factors controlling the processing of APP as targets for potential therapeutic modulation. One line of research has focused on cholesterol, which is produced in the brain (independently of the periphery) by astrocytes, then shuttled to neurons bound to apolipoprotein E (APOE) protein. APOE is encoded by the polymorphic gene *APOE* which possesses three alleles ε2, ε3, and ε4 [4], with the strongest genetic risk factor for sporadic AD being the ε4 allele of APOE [5]. Cerebral levels of cholesterol are elevated in AD and it is known to accumulate in amyloid plaques [6–10]. Additionally, APP processing occurs preferentially in cholesterol-enriched domains of the plasma membrane named lipid rafts [11, 12]. We previously showed that an increase of cholesterol in the plasma membrane triggers relocalization of APP-BACE1 complexes in lipid rafts and their clathrin-dependent internalization in enlarged endosomes, leading to increased APP processing and secretion of Aβ40 and Aβ42 [13–16]. Cholesterol has been also described as a positive regulator of BACE1 and γ-secretase, the latter enzyme cleaving the βC-terminal fragment (βCTF) resulting from the processing of APP by BACE1 [17, 18]. Reciprocally, APP regulates cholesterol homeostasis by transcriptional regulation of the key enzyme for cholesterol synthesis enzyme 3-hydroxy-3-methyl-glutaryl-coenzyme A reductase [19]*.*

**Fig. 1:**
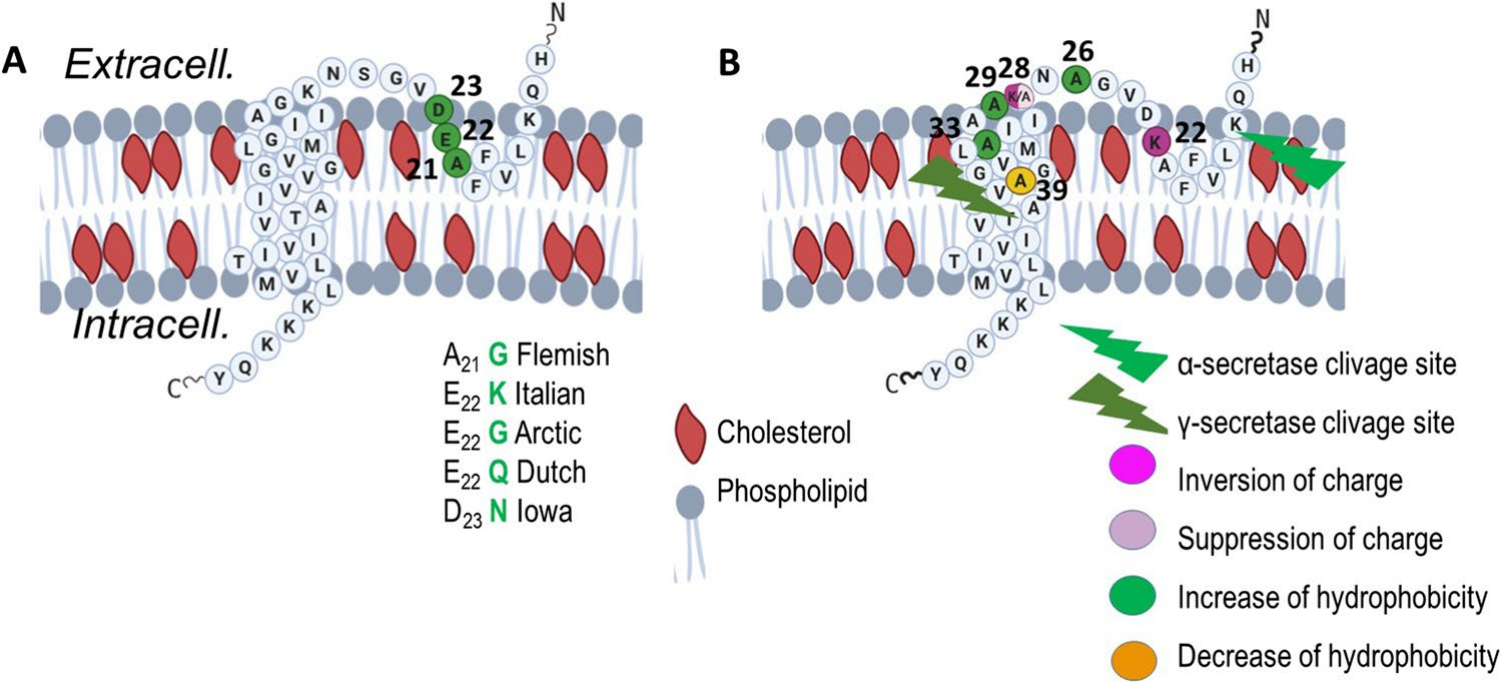
Schematic diagram of the juxta- and the transmembrane regions of APP CTFβ within the membrane, inspired from (22). Cholesterol is highlighted in red. (**A**) Amino acid sequence of the APP^WT^ and mutations from Familial Alzheimer’s disease (FAD) cases. (**B**) Indication of the mutations produced in the cholesterol-binding site (CBS) shown at positions 22, 26, 28, 29, 33, and 39. Numbering according to Aβ. Various colors indicate the type of changes in the mutants

Molecular simulation and physicochemical characterization have shown that the Aβ5–16 segment binds to the ganglioside GM1, while the Aβ22–35 segment is linked to cholesterol in the bilayer, directing the partial insertion of the peptide into the lipid raft [20, 21]. In addition, structural studies using nuclear magnetic resonance (NMR) identified a cholesterol-binding site (CBS) in the transmembrane segment forming a flexible curved α-helix in the juxtamembrane domain of the βCTF [22–24] (Fig. 1). Molecular dynamic simulations found that the APP transmembrane region and particularly the GxxxG dimerization motif was not sufficient for binding to membrane cholesterol, which also required the APP juxtamembrane segment [25]. Two helical secondary structures in the βCTF fragment of APP were identified in lysophospholipid micelles [23]. First, a R-helix that includes the transmembrane domain (TMD) terminated by three consecutive lysines. Second, a short R-helical segment (F19 through E22 in the Aβ sequence) which is located in the extracellular domain, three amino acids after the site of α-secretase cleavage (at K16 in the Aβ sequence) until the start of the TMD. This latter domain is a short reentrance loop located in a juxtamembrane region.

To clarify how the CBS regulates APP processing, we produced seven single mutants in the APP^751^ protein either in the juxtamembrane region at aa positions 22, 26, and 28 of the Aβ sequence (positions 674, 678, and 680 of APP^751^) that exhibited the greatest chemical shift perturbation in response to cholesterol in NMR studies [23] or in the TMD at aa positions 29, 33, and 39 (positions 681, 685 and 691 of APP^751^) involved in APP dimerization and orientation [26]. In addition, two double mutants, 26/28 and 29/33, were constructed. We show that most mutations triggered a reduction of Aβ40 and Aβ42 secretion from transiently transfected HEK293T cells. Only the K to A mutation at position 28 of Aβ in the APP sequence (APP^K28A^) produced shorter Aβ peptides and was insensitive to the level of plasma membrane cholesterol, in distinction to APP^WT^ [13, 16]. Since shorter Aβ sequences are known to be less aggregation-prone and toxic than the Aβ40 and Aβ42 peptides found in amyloid deposits [27, 28], targeting the juxtamembrane CBS-containing region of APP (K28A) could be a novel therapeutic option for inhibiting cholesterol-binding and hence reducing the production of toxic Aβ species.

## Results

### Production of APP Mutants of the Cholesterol-Binding Site (CBS)

Starting from the structural study that characterized the CBS on APP [23] (Fig. 1A), we used site-directed mutagenesis to produce point mutations in the APP^751^mCherry plasmid. In total, seven mutants were generated in the juxtamembrane region at positions 22, 26, and 28, and in the transmembrane region at positions 29, 33, and 39 of the Aβ sequence (positions 674, 678, and 680, and positions 681, 685, and 691 of APP^751^) (Fig. 1B). In addition, we produced two double mutants at juxtamembrane positions 26/28 and at transmembrane positions 29/33 (Fig. 1B). Point mutations corresponded to either inversion of charge of the mutated amino acid such as in the E22K and the K28E mutants, suppression of charge as in the K28A mutant, decrease of hydrophobicity as in the V39A mutant or increase of hydrophobicity as in the S26A, G29A, and G33A mutants (Fig. 1B). These changes of either charge and/or hydrophobicity at these positions have been shown by NMR to alter the cholesterol-binding properties [22]. All plasmids were sequenced and the mutations confirmed.

### Dosage of Aβ Peptides Produced by HEK293T Cells Transiently Transfected with APP Mutants of the CBS

**Fig. 2:**
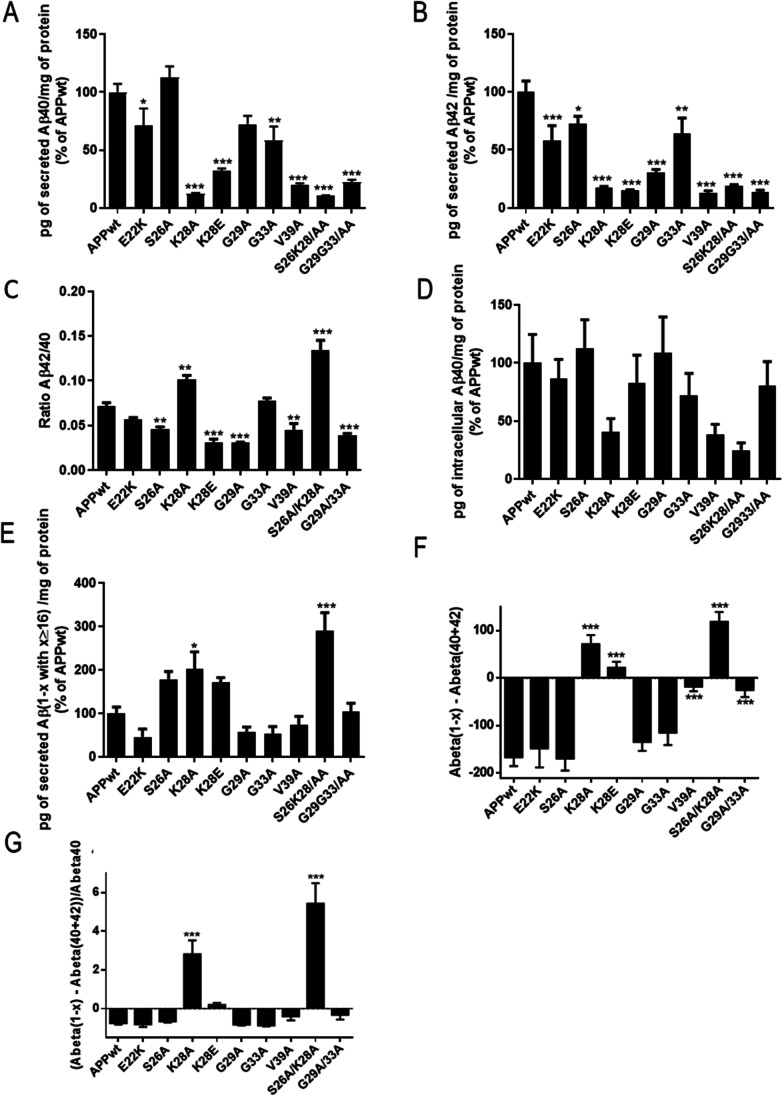
Mutations in the CBS modulate Aβ secretion in transiently transfected HEK293T cells without accumulation in the intracellular compartments. (**A**, **B**) MSD assay of extracellular Aβ40 and Aβ42 levels respectively, on transiently transfected HEK293T with different mutants of the APP CBS. The results are normalized with the amount of intracellular proteins determined by Bradford assay and represented as a percentage of Aβ produced by APP^wt^. (**C**) Calculated Aβ42/Aβ40 ratio from A-B. (**D**) MSD assay of intracellular Aβ40 levels on transiently transfected HEK293T with different mutants of the APP CBS. (**E**) ELISA (IBL kit) for Aβ(1-x with x ≥ 16) extracellular levels on HEK293T transiently transfected with the different APP mutants. (**F**) Aβ(1-x) minus Aβ40 and Aβ42 levels from A, B, and E. (**G**) Ratio Aβ(1-x) minus Aβ40 and Aβ42 over Aβ40. Results are normalized with the amount of intracellular protein determined by Bradford assay and normalized to percentage of Aβ produced by transiently transfected HEK293T cells with APP^wt^. Numbering according to Aβ. (Statistics: one way ANOVA + Dunnett’s vs APP WT mean ± SEM, *p < 0.05, **p < 0.01, ***p < 0.001, comparison with APP.^wt^, 3 independent cultures with 2 to 3 replicates per culture 6 < *n* < 9; (**A**) F(9, 70) = 25.36 *p* < 0.000; (**B**) F(9, 70) = 20.25 *p* < 0.0001; (**C**) F(9, 71) = 44.68 *p* < 0.0001 Fig. 2E; (**D**) F(6, 60) = 1.981 *p* = 0.0574; (**E**) F(9, 32) = 11 *p* < 0.0001); **(F**) F(9, 72) = 23.23 ***p < 0.0001); (**G**) F(9, 71) = 24.84 ***p < 0.0001)

We used the Meso Scale Discovery multiplex ELISA for dosage of Aβ38, Aβ40, and Aβ42 with capture antibodies recognizing the C-terminal part of the Aβ sequence (neoepitope ending at amino acid 38, 40, 42, respectively) and a detection antibody binding to the N-terminal part of Aβ [4–9]. All mutations produced were localized outside the epitopes of both the capture and the detection antibodies. As described previously, we found that the levels of Aβ40 secreted by naïve HEK293T cells were higher than the levels of secreted Aβ42 (Supplementary Fig. 1) as suggested earlier [18, 29]. We found that mutations at positions 28 and 39 strongly reduced the secretion of Aβ40 and Aβ42 as compared with APP^WT^, while the effect was weaker with mutations at positions 22, 26, 29, and 33 (Fig. 2A, B). Secretion of Aβ38 was undetectable with all transfected plasmids. We calculated the ratio Aβ42/40 with all constructs and found that the highest ratios were obtained with the K28A and S26A/K28A mutants (Fig. 2C). However, this result should be considered with caution since the levels of Aβ40 and Aβ42 with this mutant are both very low. We then asked whether the large decrease in Aβ40 and Aβ42 secretion by HEK293T cells transiently transfected with mutated APP^751^ was due to an accumulation of intracellular peptides or to differences in APP processing. We used the MSD assay to measure intracellular Aβ38, 40, and 42. In Fig. 2D, we found no intracellular accumulation but rather less Aβ40 in HEK293T cells transiently transfected with the mutants at positions 28 and the double mutant 26/28 than with the APP^WT^. Levels of Aβ42 and Aβ38 were undetectable.

We thus suspected that mutants could produce Aβ peptides shorter than Aβ40, not detected with the MSD Multiplex assay that can only quantify full length Aβ38, Aβ40, and Aβ42. We thus used the IBL ELISA with a capture antibody that targets the N terminus of Aβ peptides and a detection antibody raised against Aβ11-28 of Aβ, able to measure shorter Aβ peptides (Aβ1-x with x ≥ 16) in addition to Aβ38, Aβ40, and Aβ42. Using this IBL kit, we found that only the K28A mutant and the double mutant S26A/K28A secreted significantly more Aβ1-x peptides with x ≥ 16 (Fig. 2E). As the secretion of Aβ40 and Aβ42 was reduced using these mutants (Fig. 2A, B), we concluded that mutants at position 28 secreted shorter peptides within the range Aβx-16 to Aβx-42. We evaluated the actual levels of shorter Aβ peptides by subtracting Aβ40 and Aβ42 to Aβ1-x, even though Aβ40 and Aβ42 measures obtained with the MSD and the IBL systems using different antibodies and detection techniques are not strictly comparable. Nevertheless, using this proxy, we confirmed that only K28 mutants produced higher levels of shorter Aβ peptides (Fig. 2F). We also calculated the ratios of shorter Aβ peptides over Aβ40 and again found that only the K28A mutant and the double mutant S26A/K28A showed significant high ratio (Fig. 2G).

Altogether, we showed that mutating the CBS of APP at positions 28 of the Aβ sequence into an alanine (change of charge) but not a glutamic acid (inversion of charge) shortened the size of Aβ peptides produced while mutations at positions 22, 26, 29, 33, and 39 decreased the amount of secreted Aβ peptides ranging from Aβ16 to Aβ42. The double mutant (positions 26 and 28) showed increased production of shorter Aβ peptides, suggesting that the K28A mutation predominated (Fig. 2E).

### Processing of APPWT and Mutants

**Fig. 3:**
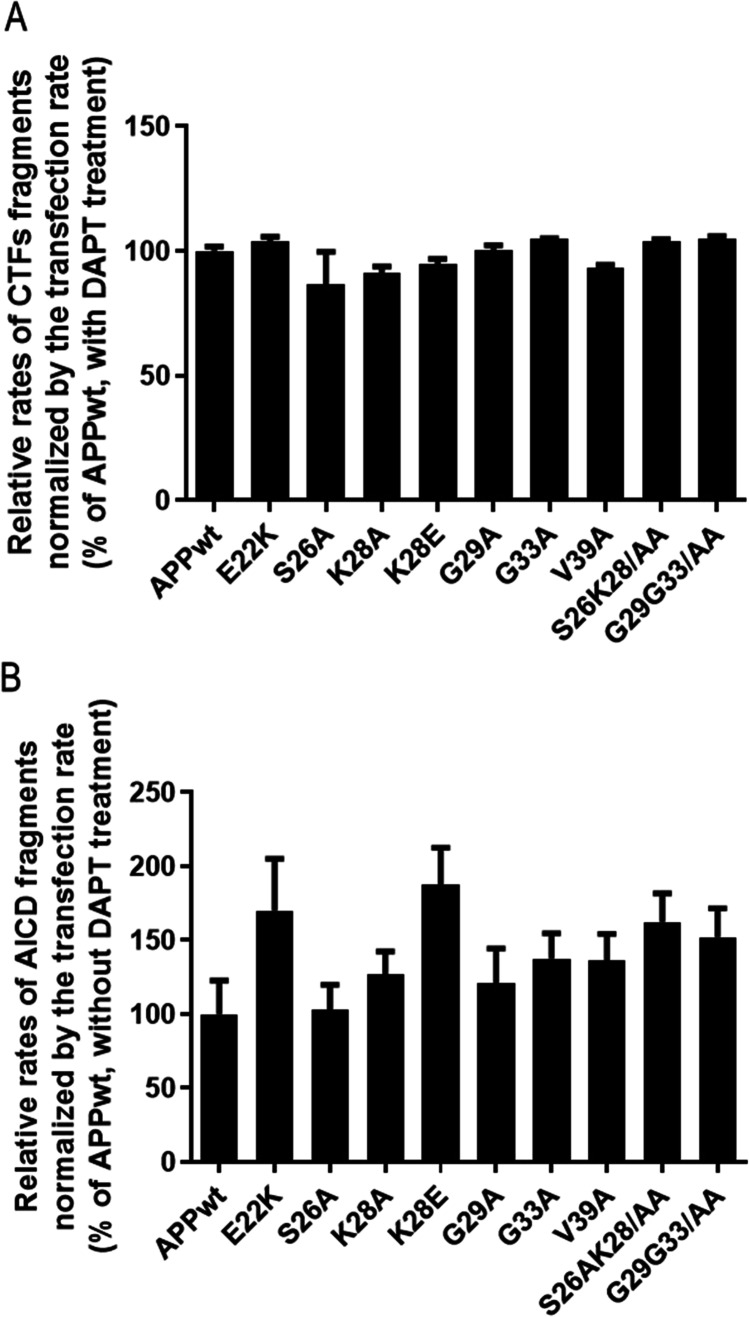
Effect of mutations in the CBS on the activity of α, β, and γ-secretase in transiently transfected HEK293T. (**A**) Relative levels of CTFs from western blots of HEK293T cell lysates transiently transfected with APP^wt^ and mutants. (**B**) Relative levels of AICD from western blots of HEK293T cell lysates transiently transfected with APP.^wt^ and mutants (Statistics: one way ANOVA + Dunnett’s vs APP WT mean + / − SEM, 3 independent cultures with 4 < *n* < 6)

We then characterized the processing of APP mutants by the α-, β-, and γ-secretase by quantifying the levels of full-length APP, C-terminal fragments (βCTF), and AICD (APP intracellular domain) in the presence or absence of the γ-secretase inhibitor, the dipeptide DAPT. In the presence of DAPT, production of AICD is inhibited, thus allowing quantification of βCTFs reflecting the activity of β-secretase only (Fig. 3A and Supplementary Fig. 2). In the absence of DAPT, the activities of α-, β-, and γ-secretases are measured through the production of AICD (Fig. 3B). Using western blot quantification, we showed that the levels of full length APPmCherry did not vary between transfections (data not shown). Moreover, CTFs and AICD were unchanged with all mutants compared to APP^WT^ (Fig. 3A, B and Supplementary Fig. 2 for examples with K28A and K28E mutants). Overall, α-, β-, and γ-secretase activities did not appear modified by CBS mutation.

### Subcellular Localization of APPWT and APP Mutants

**Fig. 4:**
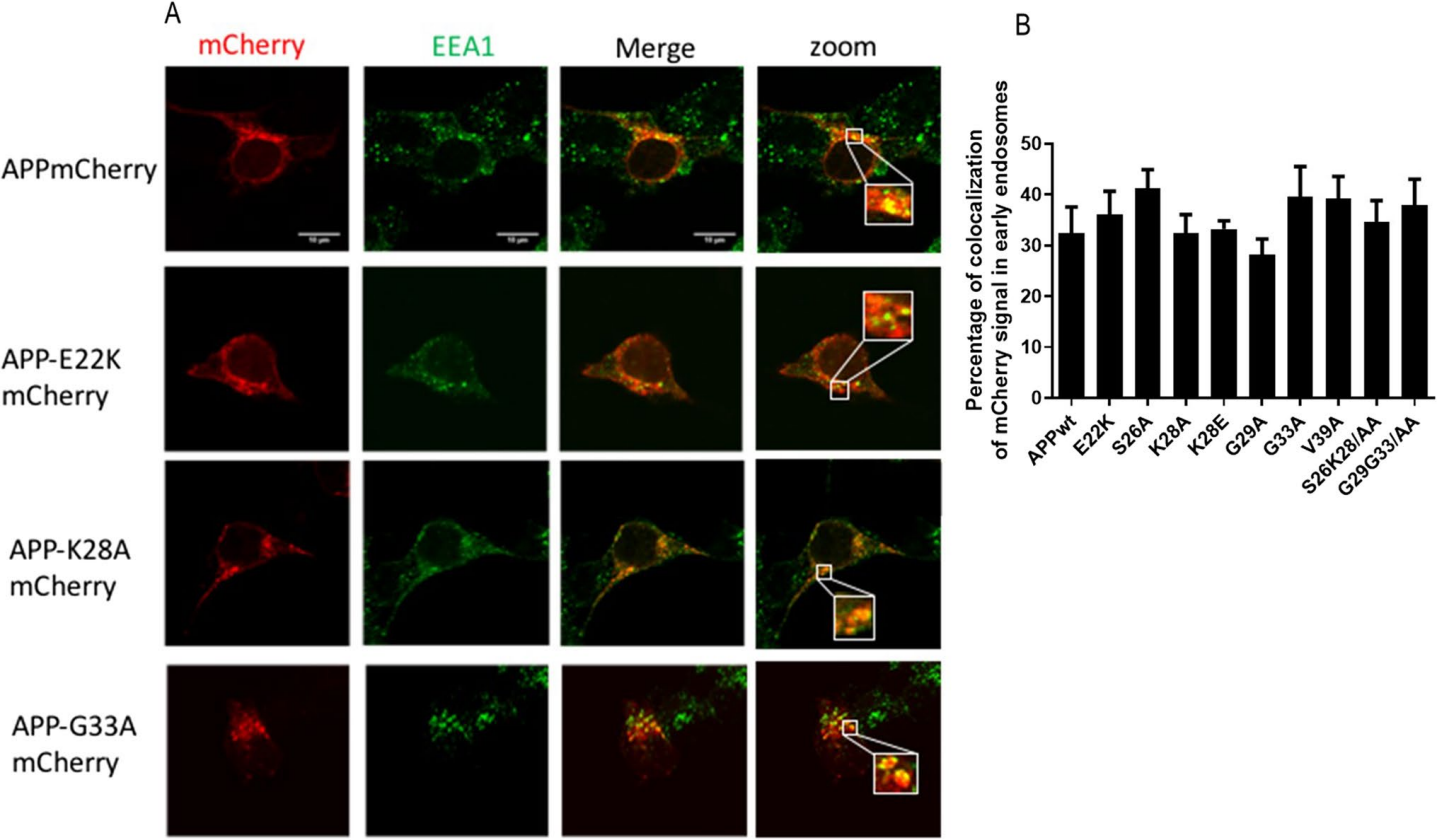
Effect of CBS mutations on endosomal localization of APP-mCherry. (**A**) Representative images of HEK293T cell transiently transfected with APP^wt^mCherry, APP^E22K^mCherry, APP^K28A^mCherry, and APP^G33A^ mCherry mutants (red), fixed after 24 h and immunolabelled with an antibody against EEA1 (green). (**B**) Percentage of colocalization of the two fluorescent signals mCherry and Alexa488 was quantified for all mutants from single stack of images acquired on the confocal microscope. The values are given as mean ± SEM. Statistical differences were analyzed one-way ANOVA + Dunnett’s (vs APP^wt^), 7 < *n* < 10 cells/experiment

Since APP processing occurs in the endolysosomal compartment, we compared the percentage of colocalization of APP^WT^-mCherry and mutants APP-mCherry with an anti-EarlyEndosomeAntigen1 (EEA1) antibody in transiently transfected HEK293T cells. Figure 4A shows representative images with APP^wt^, APP^E22K^, APP^K28A^, and APP^G33A^. The percentage of colocalization of mCherry signal in the endosomal compartment (Fig. 4B) showed that mutants APP localized in the early endosomal compartment similarly to APP^WT^, suggesting that subcellular localization of APP^WT^ and APP mutants in early endosomes was comparable.

### Effect of Cholesterol Treatment of Stable HEK293T Clones Expressing APPWT and APP^K28A^ on Aβ Peptides Production Using MSD Dosage and Mass Spectrometry

**Fig. 5:**
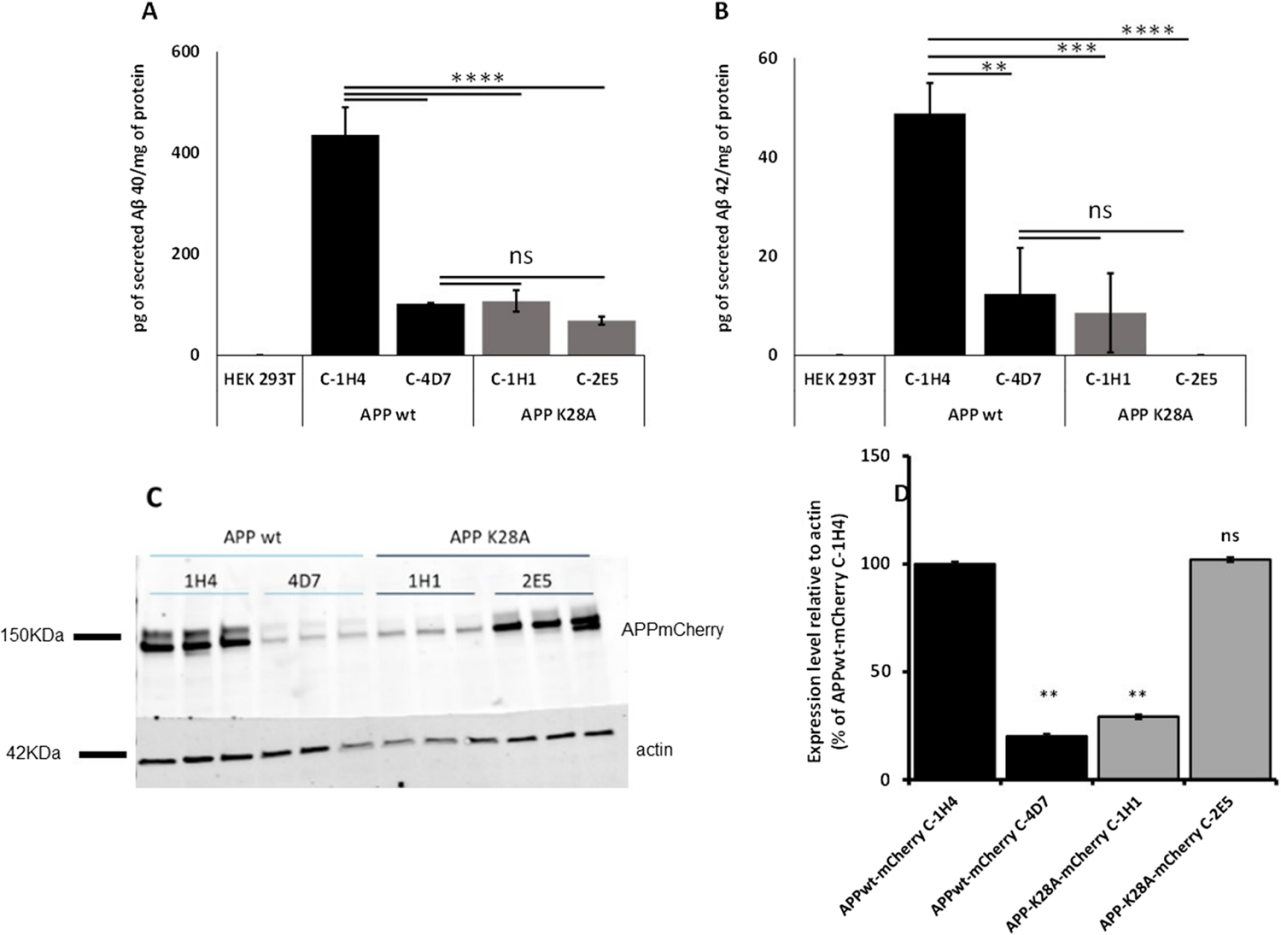
Characterization of HEK293T clones stably expressing APP^WT^mCherry and APP.^K28A^mCherry. **A**, **B** MSD assay for secreted Aβ40 and Aβ42 respectively. No Aβ40 and 42 were detected in the HEK293T untransfected control clones. One-way ANOVA Tukey’s multiple comparisons (*n* = 3, mean ± standard error). **C** Western blot analysis of mCherry expression levels of clones stably expressing APPwt-mCherry and APPK28A-mCherry were detected with an anti-mCherry antibody. D Expression levels of APPmCherry were quantified using ImageJ. Expression levels relative to actin were normalized to the APPmCherry level of each clone. Statistics: two-way ANOVA (*n* = 3, mean ± standard error). ****p < 0.0001***p < 0.001**p < 0.01

We produced HEK293T clones stably expressing APP^WT^ and APP^K28A^ and selected those which expressed high and similar levels of the protein APP-mCherry (clones 1H4 and 2E5, Fig. 5A-D). We confirmed that clone 2E5 expressing APP^K28A^ produced significantly less Aβ40 and Aβ42 peptides as compared to clone 1H4 that expressed APP^WT^ (Fig. 5A-B). We also observed that the levels of mature APP were lower with APP^K28A^ mutant (Fig. 5C).

**Fig. 6:**
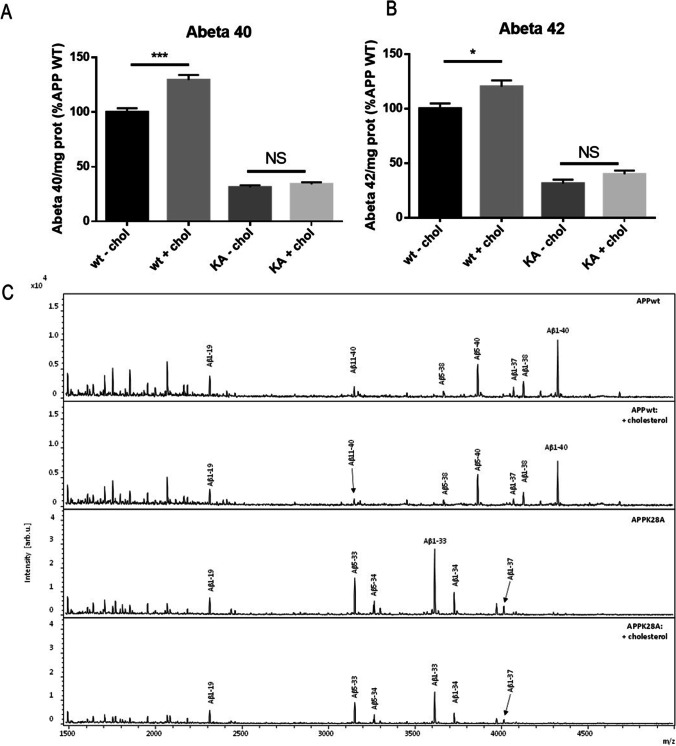
The K28A mutation in the CBS of APP abolishes the modulation of Aβ secretion by cholesterol increase at the plasma membrane. MSD assays for secreted Aβ40 (**A**) and Aβ42 (**B**) levels from HEK293T clones stably expressing APP^WT^ and APP^K28A^ in the absence and in the presence of additional methyl-β-cyclodextrin (MBCD) loaded with cholesterol. The results are normalized with the amount of intracellular proteins determined by Bradford assay and represented as a percentage of Aβ produced by APP^WT^ in the absence of MBCD loaded with cholesterol. (Test two-way ANOVA, 4 independent experiments/culture with 2 < *n* < 6; *p < 0.05, ***p < 0.001). (**C**) MALDI mass spectra of APP^WT^ and APP^K28A^ both treated with cholesterol (+ cholesterol) and untreated (− cholesterol). An Aβ pattern difference between APP^WT^ and APP^K28A^ is observed, with AβX-37/38/39/40 present in WT while AβX-33/34 peptides are the most abundant in APP^K28A^

In order to confirm that K28A mutation in APP changes the profile of Aβ peptides secreted from longer towards shorter size, we analyzed the supernatants of HEK293T clones stably expressing APP^WT^ and APP^K28A^ using MALDI-TOF–MS and LC–ESI–MS and -MS/MS. A different Aβ peptide pattern was observed between APP^WT^ and APP^K28A^ samples (Fig. 6C). In APP^WT^ samples, the full length Aβ1-40 is present, together with several N-truncations of Aβx-37, -38, -39, and -40, while these peptides are absent in the APP^K28A^ samples, where Aβx-33 and Aβx-34 are the major forms identified. A full list of the peptides identified by LC–ESI–MS/MS is shown in Supplementary Fig. 3.

We then analyzed the effects of cholesterol on the APP mutant K28A (APP^K28A^) which showed the most contrasted profile of Aβ secretion. We wanted to test whether the addition of cholesterol in the plasma membrane, known to trigger APP processing and Aβ production, was able to do so in cells expressing the APP^K28A^ mutant. We chose cholesterol addition instead of cholesterol retrieval from the plasma membrane since we anticipated that an increase of Aβ40 and Aβ42 secretion would be easier to detect than a decrease from APP^K28A^ clones secreting low levels of Aβ40 and Aβ42. HEK293T clones stably expressing APP^WT^ and APP^K28A^ were treated with 1.4 mM methyl-β-cyclodextrin (MBCD) loaded with cholesterol as described previously inducing respectively 11% and 15% increase of membrane cholesterol concentration (data not shown). While cholesterol increase at the plasma membrane raised Aβ40 and Aβ42 secretion in APP^WT^ expressing clone, APP^K28A^ clone expressing the CBS mutant was insensitive to cholesterol changes (Fig. 6A, B). In addition, MALDI analyses, although not quantitative, showed that cholesterol treatment did not change the profiles of Aβ peptides secreted by cells expressing either APP^wt^ or APP^K28A^ at a normal plasma membrane cholesterol level (Fig. 6C and Supplementary Fig. 3). In order to confirm that the observed effects of cholesterol were not due to the cleavage of endogenous APP, we treated untransfected HEK293T cells with cholesterol. We found that the levels of secreted Aβ40 and Aβ42 by naive HEK293T cells were tenfold lower than with HEK293T cells transiently transfected with APP^wt^ and more than 20 times lower than with HEK293T stable clones expressing APP^wt^ (Supplementary Fig. 1 and Figs. 2 and 5). We also did not find any significant effect of cholesterol on these low levels of secreted Aβ (Supplementary Fig. 1).

### Binding of Aβ-Derived Peptides to Membranes of Exosomes Purified from HEK293T Cells

**Fig. 7:**
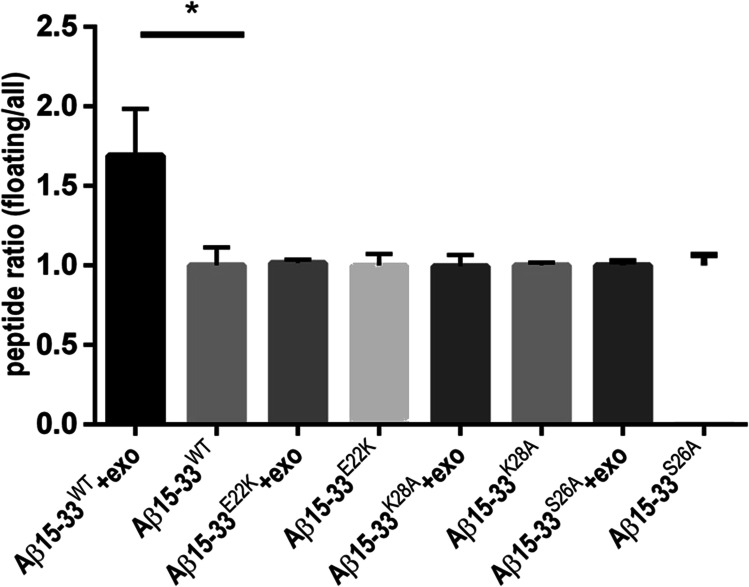
Effect of mutations of the CBS in the juxtamembrane segment of APP on their binding to exosomes. Evaluation of the amount of biotinylated peptides floating in an 11 mL sucrose gradient after incubation with and without exosomes and ultracentrifugation. Biotinylated peptides were quantified by ELISA assay using HRP-streptavidin (*n* = 3). Ratio of bound peptides corresponds to biotin signal in the first 4 fractions (0 and 10% sucrose) divided by the total signal of the 11 fractions (0 – 10 – 50 and 60% sucrose) (mean ± SD, *p < 0.05, two-tailed *p* value *n* = 3)

Next, we wanted to test whether mutations of the CBS in the juxtamembrane segment of APP could change the interaction with lipid bilayers formed by natural membranes. Three peptides corresponding to the juxtamembrane region of APP (positions 15 to 33 on Aβ) were synthetized with a linker and a biotin. In addition to Aβ15-33^WT^, we synthetized Aβ15-33^K28A^, Aβ15-33^E22K^, and Aβ15-33^S26A^ peptides. We then assessed binding of these synthetic peptides to exosomes known to have membranes enriched in cholesterol playing a crucial role in the formation and release of exosomes [30, 31]. Exosomes secreted by untransfected HEK293T cells were purified by ultracentrifugation and incubated with the three peptides for 1 h at 37 °C. Exosomes had mean size of 170 nm by Dynamic Light Scattering and were positive for CD63 and Alix as expected (Supplementary Fig. 4). Peptides bound to exosomes were separated on a sucrose gradient and quantified by luminescence with streptavidin coupled to HRP. Figure 7 shows that Aβ15-33^WT^ bound specifically to exosomes while Aβ15-33^E22K^, Aβ15-33^K28A^, and Aβ15-33^S26A^ did not, suggesting that mutations in the CBS alter the binding of Aβ15-33 peptides to cholesterol containing natural membranes.

## Discussion

Our previous work demonstrated that membrane cholesterol is an important factor modulating the processing of the transmembrane protein APP and hence the production of toxic Aβ peptides. We found that transient increase of cholesterol at the plasma membrane of non-neuronal and neuronal cells induces a rapid relocalization in lipid rafts of APP and the β-secretase BACE1, the first APP-processing enzyme of the amyloid pathway [14, 15]. This was followed by a rapid internalization of APP-BACE1 complex in endosomes with larger size and an increase of Aβ40/42 secretion [14–16].

In the present work, we demonstrate that mutations in the transmembrane or in the juxtamembrane regions of APP involved in cholesterol-binding differentially regulate APP processing. Point mutations in the APP transmembrane domain modulating the hydrophobicity of specific residues at position 29 (G29A), 33 (G33A) involved in the GxxxG dimerization motifs, and 39 (V39A) of the Aβ sequence lead to a reduction of the secretion of all Aβ peptides detected (Aβ40 and Aβ42 as well as shorter Aβ1-x with x ≥ 16 peptides), which was not due to any accumulation of intracellular Aβ peptides. In addition, the double mutation G29A and G33A provided more dramatic and cumulative effects on Aβ secretion. The G29 position was found to be critical for γ-secretase processivity, mimicking γ-secretase modulators by lessening toxic Aβ peptides [32, 33].

In contrast, we showed that point mutations in the APP juxtamembrane domain had different effects on Aβ secretion. While the E22K mutant produced less Aβ40 and/or Aβ42 and Aβ1-x with x ≥ 16, the K28A, K28E, and S26A mutants showed lower levels of Aβ40 and/or Aβ42 but higher levels of Aβ1-x with x ≥ 16 peptides that were statistically significant only with the K28A mutation. Interestingly the secreted Aβ peptide profile from the double mutant S26A and K28A showed a cumulative effect of K28A and S26A mutations. Two important residues from the juxtamembrane segment namely E22 and D23 have been found to control pH-dependant binding of cholesterol to APP [34]. At low pH, such as in the endosomal compartment, E22 and D23 are neutral and bind cholesterol thereby allowing APP processing by the β- and γ-secretases while a change of charge as in the E22K mutant alters this processing as seen herein. This glutamic acid at position 22 of Aβ is mutated in several dominant familial cases of AD (FAD) that show low levels of Aβ peptides. As mirrored here in cellular models, the Italian mutation E22K produces low levels of Aβ42 in the brain of affected individuals with prominent cerebral amyloid angiopathy and a lack of neurofibrillary tau and neuritic plaques [35, 36]. Families carrying the E22Δ deletion also showed markedly reduced levels of Aβ38, Aβ40, and Aβ42 that are less degraded into smaller Aβ peptides but form synaptotoxic Aβ oligomers [37]. Other pathological mutations at position E22 have been identified and show changes in Aβ formation. Arctic mutation carriers (E22G) have lower levels of Aβ40 and Aβ42 while Aβ protofibrils are increased [38]. Individuals carrying the E22Q Dutch mutation show hereditary cerebral hemorrhage with characteristic cerebral amyloid angiopathy and an increased formation of oligomeric and fibrillar Aβ [36]. In addition, the E22A mutation in iPSC-derived neurons was associated with lower levels of Aβ40 and Aβ42 mirroring our own study [39]. Interestingly, the E22A mutant was not sensitive to the effects of atorvastatin, a cholesterol-lowering drug. Indeed atorvastatin did not increase Aβ42 secretion in mutated neurons while it still decreased the pT231Tau/tTau ratio as in the non-mutated iPSC-derived neurons, supporting the hypothesis that the effects of cholesterol on Aβ and tau are regulated through two different pathways [39]. In addition, these data confirm that E22 located in the predicted CBS is involved in cholesterol effects. Similarly, we found that binding of the corresponding synthetic mutated peptides derived from the juxtamembrane region of Aβ (Aβ15-33^E22K^) to lipid bilayers formed by cholesterol-rich natural membranes was largely decreased as compared to Aβ15-33^WT^ peptide. This change of interaction could induce the juxtamembrane loop to unbind cholesterol from the external leaflet of the plasma membrane. Indeed, it was shown previously that the Aβ22–35 region is linked to cholesterol in the lipid bilayer [40]. In Fig. 1, we depict equal levels of membrane cholesterol in the two leaflets. However, the exact ratio of cholesterol between outer and inner leaflets has not been definitively clarified [41] with several authors suggesting that there could be ten times more cholesterol in the outer leaflet than in the inner leaflet [42, 43]. This later property could favor the binding of the Aβ juxtamembrane region to the enriched cholesterol leaflet.

The profile of Aβ secreted by the K28A mutant showed a decrease of Aβ40 and Aβ42 and an increase of Aβ1-x with x ≥ 16, suggesting that shorter Aβ peptides are produced. In addition, other mutants at positions S26 and K28 namely S26L and K28S were also found to produce large decrease of Aβ40/42 secretion [44]. The most distinctive and striking effects were obtained with the K28A mutant in transiently transfected cells and in stable clones expressing this mutant as compared to wt APP. Using MS methods, we confirmed that the K28A mutant produced mostly Aβ33 and Aβ34 peptides with no detectable levels of Aβ40 or Aβ42, corroborating and amplifying a previous study [45]. In another study, the K28E mutant had a more contrasted effect with Aβ37 being the most abundant form [46]. Here, the K28E mutant produced higher amounts of Aβ (1 × with x ≥ 16) as compared to WT, although the increase was not statistically significant (Fig. 2E). The difference in Aβ length could be due either to changes in γ-secretase activity (either the first ε cleavage producing Aβ49 and AICD 50–99 or Aβ48 and AICD 49–99, or the sequential cleavages relying on γ-secretase carboxypeptidase-like trimming activity releasing tri- and tetrapeptides and Aβ46 Aβ43 and Aβ40 from Aβ49 and Aβ45, Aβ42, and Aβ38 from Aβ48). Here we show that α- and β- secretase activities are not affected by mutations (levels of CTF fragments are unchanged by mutations in the presence of DAPT, a γ-secretase inhibitor, Fig. 3A). Since AICD levels are also unchanged by mutations (Fig. 3B), we thus concluded that γ-secretase activity is not altered by mutations. However, we cannot rule out that either the first cleavage or the trimming activity of γ-secretase is different between APP^wt^ and mutants. If the first cut were different (AICD 33–99 or AICD 34–99 instead of AICD 49–99 or AICD 50–99), then the size of AICDmCherry fragments would vary from 290 to 275 aa, a difference of about 2kD that would not be detected on a 16.5% polyacrylamide gel. However, Kukar et al. [45] showed, using γ-secretase in vitro assay and MS, that the K28A mutation does not produce AICD fragments longer than 50–99 thus suggesting that there is no shift in the ε cleavage site that would give rise to shorter Aβ peptides and longer AICD fragments (Supplementary Fig. 1 of [45]). It is thus more likely that the trimming activity of γ-secretase is different with the K28A mutant, as suggested also by Devkota et al. [47] for mutations at position 42 to 48.

Single-particle cryo-EM structure of mutated γ-secretase cross-linked to mutated APP C83 revealed that C83 binds to the γ-secretase complex then inducing a conformational change in the three residues of the TM helix (Thr^47^, Leu^48^, and Val^49^ of the Aβ sequence) [48]. This structural change allows cleavage either between 47 and 48 or between 48 and 49 thus yielding either Aβ48 or Aβ49 respectively. This first cleavage by γ-secretase would not be different in the K28A mutant as shown by Kukar et al. [45]. This lysine appeared by cryo-EM at the edge of the TM helix and just next to the disordered fragment from N27 to D23 in the EM density map. Indeed Val^24^ was mutated for the crosslinking to the γ-secretase. The E^22^ to L^27^ fragment forms the N-terminal loop of C83 fragment. Because C99 is 16 aa longer, the structure could still be different. Nevertheless, since both the activity of γ-secretase is not altered by CBS mutations as shown here and the first ε cleavage of APP^K28A^ by γ-secretase occurs at the same position as in APP^wt^, CBS is likely to affect the trimming activity of the γ-secretase, giving rise to shorter Aβ peptides as shown with the K28A mutant [45]. It was concluded that lysine at position 28 anchors the Aβ sequence at the juxtamembrane thus limiting the accessibility to the stationary transmembrane γ secretase. Here we suggest that mutating this lysine would additionally suppress cholesterol-binding, “de-stapling” the Aβ48/49 peptides anchored at the juxtamembrane thus allowing entry in the transmembrane γ secretase active site and producing mainly Aβx-33 and Aβx-34 peptides. Previous molecular dynamics simulations have shown that K28A mutation reduces intrapeptide hydrophobic interactions between E22/D23 and K28 [49] and increases helix content while decreasing β-sheet and hydrophobic contacts and electrostatic interactions [50, 51]. Recent cryo-EM analysis of Aβ filaments from human post-mortem brain samples from patients with AD showed that K28 from one protofilament interacted with the C-terminal carboxyl group of Aβ42 from the other protofilament, and vice versa [52]. The K28A mutation would thus not only reduce the interaction with cholesterol but also decrease intrapeptide interactions.

According to our previous work [14–16] and the data presented here, a transient elevation in membrane cholesterol increase triggers APP^WT^ processing and Aβ40/42 secretion. We found the APP^K28A^ mutant to be insensitive to the effect of membrane cholesterol increase on Aβ secretion with no change in the levels nor the profile of Aβ peptides as determined using sensitive and quantitative ELISAs and complementary MS informing on the size of peptides secreted. We cannot exclude that the more precise quantification of Aβ33 or Aβ34 would reveal differences between cells expressing APP^K28A^ as compared to APP^WT^ following cholesterol treatment. A specific Aβ33 and Aβ34 ELISA would answer this question. Alternatively, increased endocytosis of APP^WT^ following cholesterol treatment and leading to higher production of Aβ peptides [14–16] could be affected by the K28A. Figure 4 shows that subcellular localization of APP^K28A^mCherry in the endosomal compartment does not differ from APP^wt^mCherry. It remains to be shown whether higher cholesterol would be able to increase APP^K28A^ endocytosis as with APP^wt^. Nevertheless, K28 has been identified as a key aa for the interaction of C99 with cholesterol [22, 23]. Indeed, binding of the corresponding synthetic mutated engineered peptides derived from the juxtamembrane region of Aβ (Aβ15-33^K28A^) to lipid bilayers formed by natural membranes was substantially decreased as compared to Aβ15-33^WT^ peptide. Thus, the affinity of Aβ15-33^K28A^ for the cholesterol-rich membrane is reduced, therefore their tilting within the membrane and in particular within the outer leaflet is likely modified, as suggested in Fig. 6 of Ousson et al. [46]. Consequently, these mutants appear at different positions in the plasma and intracellular membranes, inducing changes in their cleavage by γ-secretase and generating shorter Aβ peptides. One limitation of our study is the lack of experiment combining simultaneously cholesterol binding to APP and APP processing that would allow to test directly the effects of cholesterol binding on the production of Aβ peptides. But so is the case of published studies.

Aβ34 is generated through degradation of Aβ40 and Aβ42, but not βCTF, by BACE1 [53, 54]. It is present in the brain of AD patients and 3xTg mice [55], and levels are elevated in individuals with mild cognitive impairments at risk for dementia, correlating with amyloid positivity and pericyte-mediated clearance [56–58]. Here we show that APP cleavage by BACE1 is not affected by the K28A mutation. Whether degradation of Aβ40^K28A^ and Aβ42^K28A^ by BACE1 is favored remains to be established using synthetic peptides and reconstituted BACE1 protein. However, this is quite unlikely since we show that the K28A mutation mostly produces Aβ33 peptides that are not degraded from Aβ34 by the matrix metalloproteases MMP-2 and MMP-9 [59].

Collectively, the present data suggest that shorter Aβ peptides produced by K28A mutation leading to altered anchoring and positioning of longer Aβ peptides (Aβ48 and Aβ49) in the plasma membrane could be due, at least in part, to changes in membrane cholesterol binding. A tight regulation of membrane cholesterol is particularly important at the presynaptic terminals of neuronal cells where APP is enriched and its expression, distribution, and processing are regulated by synaptic activity [60, 61]. Thus, together with neuronal activity, the cholesterol content of membranes and the CBS could play a critical role in regulating Aβ processing and the resulting amount, size, and toxicity of Aβ peptides.

Our observations underpin interest in the cerebral levels and actions of cholesterol as a potential target for the treatment of AD: indeed, several strategies for its modulation have yielded encouraging observations in preclinical models [62]. For example, CYP46A1 overexpression to accelerate brain cholesterol clearance reduces amyloid pathology and improves cognitive deficits in murine models for AD [63]. Intriguingly, and suggesting the broader relevance of cholesterol, in cultured neurons cholesterol has been shown not only to regulate Aβ secretion through its interaction with the APP CBS, but also tau pathology via a different mechanism involving the proteasome [39]. The present results establish a role of the juxtamembrane region of APP containing the CBS as a regulator of the size of Aβ peptides produced: this specific region could then be a novel target for inhibiting cholesterol-binding and reducing the production of toxic Aβ species. K28 has been targeted using lysine-specific molecular tweezers to inhibit assembly and toxicity of amyloid peptides [64–66]. It will be of interest to determine whether editing of this residue using CRISPR Cas9, antisense knockdown of the mutant protein, or treatment with antibodies targeting the juxtamembraneregion will alter the profile of Aβ secretion.

### Experimental Procedures

#### Plasmid and Reagents

The APP^751^ plasmid was a kind gift from Dr. Frederic Checler (IPMC, Valbonne, France). The APP-mCherry plasmid was generated by introducing the APP^751^ sequence in the pmCherry-N1 vector (Clontech) at the *XmaI/AgeI* restriction site. MβCD-cholesterol complex, bovine serum albumin (BSA), sucrose, and poly-L-lysine were purchased from Sigma-Aldrich. The antibody directed against EEA1 (Early Endosome Antigen 1) was from Cell Signaling Technology. Goat anti rabbit IgG coupled to Alexa568 was from Life Technologies.

#### Peptides Synthesis

Amyloid peptides derived from the Aβ sequence (position 15 to 33) with an additional hydrophobic sequence (YEVH) linked to biotin were synthesized. Fmoc-amino acids, and 2-(6-chloro-1H-benzotriazole-1-yl)-1, 1, 3, 3,-tetra-methylaminium hexafluorophosphate (HCTU) were obtained from Novabiochem. N-biotine-NH(PEG)X-COOH were purchased at Merck and Sigma Aldrich. The resin and all the peptide synthesis grade reagents (N-methylpyrrolidone (NMP), N-methylmorpholine (NMM), dichloromethane, piperidine, trifluoroacetic acid (TFA), anisole, thioanisole, and triisopropylsilane) were purchased from Sigma.

Synthesis of the different Aβ peptides were performed on a Gyros-Protein Technologies, Inc., prelude synthesizer at a 25-µmol scale using a tenfold excess of Fmoc-amino acid relative to the preloaded Fmoc-Gly-wang-LLresin (0.33 mmol/g) or Fmoc-Ala-wang-LLresin (0.33 mmol/g). Fmoc-protected amino acids were used with the following sidechain protections: tert-butyl ester (Glu and Asp), tert-butyl ether (Ser and Tyr), trityl (His, Asn, and Gln), and tert-butoxycarbonyl (Lys). Amino acids were coupled twice for 5 min using 1:1:2 amino acid/HCTU/NMM in NMP. After incorporation of each residue, the resin was acetylated for 5 min using a 50-fold excess of a mixture of acetic anhydride and NMM in NMP. Fmoc deprotection was performed twice for 3 min using 20% piperidine in NMP, and 30-s NMP top washes were performed between deprotection and coupling and after acetylation steps.

Biotinylation of APP peptide was performed on the resin after Fmoc deprotection of the N-terminal residue, using a tenfold excess of N-biotine-NH(PEG)X-COOH and HCTU and NMM as coupling reagents (see above).

After completion, the peptidyl-resins were treated with a mixture of TFA/thioanisole/anisole/TPS/water (82:5:5:2.5:5) for 2 h. The crude peptides were obtained after precipitation and washes in cold ethyl ether followed by dissolution in 10% acetic acid and lyophilization.

Peptides were purified by reverse phase HPLC using an X-Bridge BHE C18-300–5 semi-preparative column (Waters, USA) (250 × 10 mm; 4 ml·min^−1^; solvent A, H_2_O/TFA 0.1%; solvent B, acetonitrile/TFA 0.1% using a gradient of 0–60% solvent B into A in 60 min. The purity of each peptide was checked by mass spectrometry using ESI–MS (Bruker). Lyophilized peptides were solubilized in water-acetonitrile (50%) and stored at − 80 °C.

#### Site-Directed Mutagenesis

APP-mCherry was mutated at single or double sites using the QuickChange II site directed mutagenesis kit (Agilent) following provider-issued recommendations. Parental methylated DNA was degraded following digestion with *DpnI*, and the remaining mutated DNA was transfected in competent XL1-Blue *E. Coli* (Invitrogen). Transformed bacteria were selected on Petri dishes filled with medium containing kanamycin (30 mg/mL) (Invitrogen). In total, 7 single and 2 double point mutations were produced in the cholesterol-binding site, in the juxtamembrane region of APP, at positions 22, 26, 28, 29, 33, and 39 of the Aβ sequence. Mutants are illustrated in Fig. 1B.

#### Cell Culture, Transfection, and Treatments

HEK293T cells were grown in DMEM-glutamax medium (Gibco, Thermo Fisher Scientific) supplemented with 10% fetal calf serum (Invitrogen, CA, USA) and 1% penicillin/streptomycin (Gibco) at 37 °C and 5% CO_2_. Transfection experiments were carried out on HEK293T cells with 70% confluence in OptiMEM medium (Gibco) without antibiotic using lipofectamine 2000 (Invitrogen). 0.7 μg of plasmid was diluted in 100 μL of OptiMEM medium (Gibco). In parallel, 4 μL of lipofectamine 2000 (Invitrogen) were mixed with 100 μL of OptiMEM medium. Transfection was performed according to manufacturer’s instructions (Invitrogen). After deposition of the transfection mix, the cells were placed for 4 h in the incubator at 37 °C, washed with 1X PBS to remove any trace of transfectant and then incubated with complete medium: DMEM medium supplemented with 10% fetal calf serum and 1% penicillin–streptomycin (Gibco).

Treatment with DAPT: In order to analyze the cleavage of APP-mCherry mutants by the β- and γ-secretases, 24 h after transfection, cells were treated for 16 h with N-[N-(3,5-Difluorophenacetyl)-L-alanyl]–phenylglycine T-butyl ester (DAPT) (Sigma Aldrich), an inhibitor of γ-secretase, diluted to a concentration of 5 µM in complete new medium.

Cholesterol treatment: 24 h after transfection, HEK293T cells were washed twice with DMEM medium (Gibco), treated for 30 min with 1.4 mM MβCD-cholesterol (Sigma), dissolved in DMEM medium, and then washed three times with DMEM medium.

Cholesterol was assessed separately using the Amplex™ Red Cholesterol Assay Kit from Invitrogen (Thermo Fisher Scientific).

#### Protein Extraction and Western Blot

The HEK293T cells were washed with 1X PBS and then lysed on ice using a buffer RIPA (50 mM Tris–HCl pH 8.0, 150 mM sodium chloride, 1.0% NP-40, 0.5% sodium deoxycholate, and 0.1% sodium dodecyl sulfate) (Sigma Aldrich), to which were added phenylmethylsulfonyl fluoride 100X (Sigma Aldrich) and a cocktail of inhibitors of proteases (Complete Mini, Roche). The lysates were sonicated 3 times for 5 min then stored at − 80 °C. Protein concentration of the lysates was quantified by Bradford assay (Biorad) according to the manufacturer’s instructions. Western blots were made from the cell lysates of HEK293T cells treated with DAPT. Proteins from cultured lysates HEK293T were separated in 16.5% Tris-Tricine (Biorad) polyacrylamide gels. Proteins were transferred to a polyvinylidene difluoride membrane (Biorad) at 150 V for 3 h at 4 °C. After 1 h of saturation with 10% milk, membranes were incubated overnight at 4 °C with primary antibodies directed against APP and actin β proteins diluted to 1/2000 in BSA-azide 2% solution. Membranes were incubated with fluorescent secondary antibodies (diluted to 1/10,000 in 0.05% TBS-tween solution) for 1 h under agitation at room temperature and away from direct light. The revelation and quantification of fluorescence were carried out by Odyssey’s analysis software (Set up ImageStudio CLx) (Odyssey Clx LI-COR).

#### Confocal Imaging

HEK293T cells cultured on poly-D-lysine-coated coverslips were fixed 24 h after transfection using a solution of 4% paraformaldehyde in PBS for 15 min at room temperature. Cells were first incubated with anti-EEA1 antibody (1/500, cell signaling) and then with goat anti-rabbit secondary antibody conjugated to Alexa 488 (1/1000, cell signaling). Coverslips were mounted in Fluoromount medium (Southern Biotech, AL, USA). Z-stack of cells were acquired on a Fluoview FV1000 confocal microscope (Olympus, Tokyo, Japan). Fluorescence was collected with a 60 × plan apochromat immersion oil objective (NA 1.35). The mean endosomes size and number per cell were analyzed with ICY software. Between 7 and 10 cells were analyzed.

#### Aβ38, 40, and 42 Measurements

Supernatants of HEK293T cells were collected on ice 24 h after transfection in polypropylene tubes, containing phosphatase inhibitor cocktail (Complete Mini, Roche). Supernatants were then stored at − 80 °C. Concentrations of the Aβ38, Aβ40, and Aβ42 species of β-amyloid peptide were measured by multiplex Electro-Chemiluminescence Immuno-Assay (ECLIA). Assays were performed according to the manufacturer’s instructions (Meso Scale Discovery (MSD) Meso QuickPlex SQ120). One hundred microliters of blocking buffer solution were first added to all wells to avoid non-specific binding. The plates were then sealed and incubated at room temperature on a plate shaker (450 rpm) for 1 h. Wells were then washed three times with washing buffer, and 25 μl of the standards peptides (Aβ38, Aβ40, Aβ42) (MSD) and samples were then added to the wells, followed by an Aβ-detecting antibody at 1 μg/ml labelled with a Ruthenium (II) trisbipyridine N-hydroxysuccinimide ester (MSD). This detection antibody was 6E10 (raised against the common epitope Aβ1-16 of the human peptide, therefore the 3 Aβ38, Aβ40, Aβ42) (MSD). Plates were then aspirated and washed 3 times. MSD read buffer (containing tripropylamine as co-reactant for light generation in the electrochemiluminescence immunoassay) was added to wells before reading on the Sector Imager. A small electric current passed through a microelectrode present in each well to produce a redox reaction of the Ru^2+^ cation, emitting 620-nm red lights. The concentration of each Aβ peptide was calculated in pg/ml for each sample, using dose–response curves It was then normalized by protein concentration measured by Bradford assay (Bradford).

#### Aβ (1-x) Measurements

Supernatants of HEK293T cells were collected on ice 24 h after transfection in polypropylene tubes, containing phosphatase inhibitor cocktail (Complete Mini, Roche). Supernatants were then stored at − 80 °C. Concentrations of peptides Aβ28, 40, and 42 were measured by enzyme-linked immunosorbent assay (ELISA) using the IBL Aβ(1-x) kit. This kit is a solid phase ELISA sandwich using 2 kinds of highly specific antibodies. Assays were performed according to the manufacturer’s instructions. Samples were plated in 96-well plates, containing antibodies specific for the Aβ (precoated plate: anti-human Aβ(N)(82E1) Mouse IgG). Briefly, 100 μl of sample or 100 µL of Aβ40 synthetic peptides (used as the standard range, IBL) were deposited in 96-well plates. The plates were sealed and incubated overnight at 4 °C with gentle agitation. After several washings of the plates (minimum 7), 100 μl of anti-Aβ antibody solution (mouse IgG directed against the epitope 11–28 of the human Aβ peptide, IBL) coupled to horseradish peroxidase (HRP) were deposited in the wells. The plaques were then sealed and incubated for 1 h at 4 °C with gentle agitation. The plates were then washed 9 times with a wash buffer. One hundred microliters of a solution containing a tetramethylbenzidine colorimetric agent, were deposited in the wells. After 30 min of incubation at room temperature and protected from light, a stop solution was deposited. Absorbance was measured at 450 nm (Thermo multiskan EX, Thermo-Fisher Scientific) within 30 min of depositing the stop solution. The concentration of each Aβ peptide was calculated in pg/ml for each sample, using dose–response curves. It was then normalized by the protein concentration measured by Bradford assay (Bradford).

#### Immunoprecipitation

Four μg of the Aβ-specific antibodies 6E10 (1–16, Biolegend) and 4G8 (17–24, Biolegend) were added separately to 25 μL of Dynabeads M-280 sheep anti-mouse (ThermoFisher Scientific) suspension, according to the manufacturer’s description. The washed antibody-bead complexes were combined (50 μL in total) and added to 3 ml supernatants of HEK293T cells together with 20% (v/v) Triton X-100 to a final concentration of 0.2% (m/v) and incubated overnight at + 4 °C. The beads/sample complex was transferred to the KingFisher for automatic washing (in 0.2% Triton X-100, phosphate-buffered saline (PBS), pH 7.6, and 50 mM ammonium bicarbonate) and elution in 0.5% FA (v/v). The eluate was dried down in a vacuum centrifuge pending MS analysis.

#### Mass Spectrometry

Analysis by matrix-assisted laser desorption/ionization time-of-flight mass spectrometry (MALDI-TOF–MS) was performed using an UltraFleXtreme instrument (Bruker Daltonics) in reflector mode. Prior to analysis, samples were reconstituted in 5 μl 0.1% FA/20% acetonitrile in water (v/v/v). MALDI samples were prepared using the seed layer method as previously described [67]. An average of 10,000 shots was acquired for each spectrum (2000 at a time using a random walk mode). The unused sample (3 μl) was further dried down in a vacuum centrifuge and further analyzed by nanoflow liquid chromatography (LC) coupled to electrospray ionization (ESI) hybrid quadrupole–orbitrap tandem MS (MS/MS), see below.

Analysis by nanoflow LC–ESI–MS/MS (Dionex Ultimate 3000 system and Q Exactive, both Thermo Fisher Scientific) was performed in a similar way as described previously [68, 69]. Briefly, samples were reconstituted in 7 μl 8% FA/8% acetonitrile in water (v/v/v). An Acclaim PepMap 100 C18 trap column (20 mm × 75 μm, particle size 3 μm, pore size 100 Å, Thermo Fisher Scientific) was used for online desalting and cleanup. For separation, a reversed-phase Acclaim PepMap RSLC column (150 mm × 75 μm, particle size 2 μm, pore size 100 Å, Thermo Fisher Scientific) was used. Mobile phases were 0.1% FA in water (v/v) (A) and 0.1% FA/84% acetonitrile in water (v/v/v) (B). Separation was performed at a flow rate of 300 nl/min by applying a linear gradient of 3–40% B for 50 min at 60 °C. Spectra were acquired in positive ion mode for the mass-to-charge (m/z) range 350–1800. Both MS and MS/MS acquisitions were obtained at a resolution setting of 70,000 using 1 microscan, target values of 10^6^, and maximum injection time of 250 ms. MS/MS acquisitions were obtained using so-called higher-energy collisional dissociation fragmentation at a normalized collision energy setting of 25, an isolation window of 3 m/z units, and exclusion of singly charged ions and ions with unassigned charge.

Database search, including isotope and charge deconvolution, and peak area determination, was performed with PEAKS Studio X + (Bioinformatics Solutions Inc.) against a custom-made APP database, which included the K28A-modified sequence. All suggested fragment mass spectra were validated manually. For the label-free quantitative analysis, normalization was performed for each sample by division of the raw area with the total protein content (as determined by the Bradford assay, see above). MS data are available in Supplementary data.

#### Purification of Exosomes from HEK293T Cell Culture Medium

HEK293T cells were grown in DMEM 10% FCS in T150 flasks coated with Poly-lysin. At 80% confluence, cells were rinsed in PBS 1X heated at 37 °C and resuspended in OPTIMEM without FCS and incubated at 37 °C during 24 h. Culture medium was collected in 50-mL Falcon tubes then centrifuged at 2000 g for 20 min at 4 °C. Supernatant was filtered through 0.22-µm Millipore filters and centrifuged at 100,000 g for 2 h at 4 °C. Pellets containing the exosomes were rinsed with ice-cold PBS, centrifuged again at 100,000 g for 2 h at 4 °C and resuspended in 75 µL ice-cold PBS. The size of exosomes was measured using Dynamic Light Scattering. Two microliters of Red Blood Cell exosomes as control, and 2 µL of HEK293T cells exosomes were spotted on nitrocellulose and dried. The blot was then treated with 5% milk for blocking, then stained with 1/1000 antibodies against human CD63 and Alix (Becton Dickinson and Cell Signaling) overnight. The blot was then hybridized with 1/5000 HRP conjugated antibody (anti mouse and anti-rabbit). Two microliters of HEK293T exosome solution were deposited on Formwar-coated grids. Exosomes were stained using 2% uranyl acetate for 10 min and rinsed three times in water. Grids were observed under a a Hitachi HT 7700 electron microscope operating at 70 kV (Elexience, Verrieres-le-Buisson, France).

#### Binding of Amyloid Peptides to Exosomes

Peptides (15 µL at 20 µM) were incubated with 10 µL of exosomes (cholesterol at 30 µM) in 50 µL final volume complemented with PBS at 37 °C for 1 h under agitation, in 2-mL Eppendorf tubes (allowing maximum agitation). Solutions were diluted in 400 µL PBS and deposited on a sucrose gradient in 5-mL tubes (from bottom to top: 3.6 mL 60% sucrose solution in PBS, 600 µL 50% sucrose solution and 400 µL 10% sucrose solution—the exosome + peptide mix is at the top), and centrifuged at 140,000 g for 2 h in a swinging rotor. Eleven fractions were collected: 1 to 4 of 200 µL, 5 to 7 of 300 µL, and the next 4 fractions of 1 mL. Concentrations of peptides were measured by immune-enzymatic assay (ELISA) using biotin-streptavidin-HRP detection. Assays were performed according to the manufacturer’s instructions. Briefly, 25 μl of each fraction were deposited in 96-well plates (Immunoplate, Nunc). The plates were sealed and incubated overnight at 4 °C with gentle agitation. After 3 washings of the plates in PBS, 100 µL BSA 1% in PBS was added for 1 h followed by 3 washings in PBS. Streptavidin solution coupled to horseradish peroxidase (HRP) (Sigma) in PBS (1/2500 dilution) were deposited in the wells (100 µL). The plaques were then sealed and incubated for 1 h at 4 °C with gentle agitation. The plates were then washed 3 times with 0.05% PBS-tween solution. After 30 min of incubation at room temperature with 3,3′, 5, 5′- tetramethylbenzidine (TMB Peroxidase EIA Substrate Kit, Biorad), a stop solution (H_2_SO_4_ 2 M) was deposited. Absorbance was measured at 450 nm (Varioscan) within 30 min of the deposition of the stop solution.

#### Statistical Analysis

All analyses were performed using GraphPad Prism version 6.00 for Windows. Statistical tests were two-tailed and conducted at a 5% significance level. Student’s *t* test was performed to compare mutant vs control condition, and multiple comparisons were analyzed using one-way or two-way ANOVA with Dunnett’s post hoc tests.

Data were derived from 3 independent cultures with 2 to 3 replicates per culture, except confocal imaging which was performed on 1 culture and 7 to 10 cells per condition. Data are presented as mean ± SEM.

## Supplementary Information

Below is the link to the electronic supplementary material.

Supplementary Fig. 1. Levels of Aβ40 and Aβ42 from naïve HEK293T cells in the absence and in the presence of additional methyl-β-cyclodextrin (MBCD) loaded with cholesterol. The results are normalized with the amount of intracellular proteins determined by Bradford assay and represented as a percentage of Aβ produced by HEK293T cells in the absence of MBCD loaded with cholesterol. (Unpaired t test, two tailed; 2 independent experiments/culture with 2 <n <6). (JPG 124 KB)

Supplementary Fig. 2. Representative western blots of HEK293T cell lysates transiently transfected with APP^wt^mCherry, APP^K28A^mCherry and APP^K28E^mCherry mutants treated or not with the γ-secretase inhibitor DAPT hybridized with anti-APPmCherry and actin antibodies. Quantifications are in Fig. 3. (JPG 88 KB)

Supplementary Fig. 3. Heatmap showing APP/Aβ peptides detected using LC-ESI-MS for APP^WT^ and APP^K28A^ treated (+) and untreated (-) with cholesterol. Peptide numbering refers to the Aβ sequence, where negative numbers indicate the number of positions N-terminally of the BACE1 cleavage site. The instensity (logarithmic scale) is the peak area normalised to total protein content in the respective sample. (JPG 166 KB)

Supplementary Fig. 4. Characterization of exosomes isolated from HEK293T cells. **A**: Transmission electron microscopy (TEM) representative image; B: Dotblot of red blood cells exosomes (Ex.GR) and of HEK293T exosomes (Ex.HEK) stained with anti-CD63 and anti-Alix antibodies (1/1000 dilution). (JPG 70 KB)

## Data Availability

MS data are available at 10.5281/zenodo.6274232.

## Abbreviations and Nomenclature

Aβ: Amyloid-β
AA: Amino acid
AD: Alzheimer’s disease
AICD: APP intracellular domain
APOE: Apolipoprotein E
APP: Amyloid precursor protein
CBS: Cholesterol-binding site
βCTF: C-terminal fragment
DAPT: *tert*-Butyl (2*S*)-2-[[(2*S*)-2-[[2-(3,5-difluorophenyl)acetyl]amino]propanoyl]amino]-2-phenylacetate
EEA1: Early endosome antigen1
MBCD: Methyl-β-cyclodextrin
NMR: Nuclear magnetic resonance
TMD: Transmembrane domain
