# Supplementary figures and images for: Specific Mutations in the Cholesterol-Binding Site of APP Alter Its Processing and Favor the Production of Shorter, Less Toxic Aβ Peptides
Source: Mol Neurobiol. 2022 Sep 9;59(11):7056–73. doi: 10.1007/s12035-022-03025-9 (PMC9525381; doi:10.1007/s12035-022-03025-9)

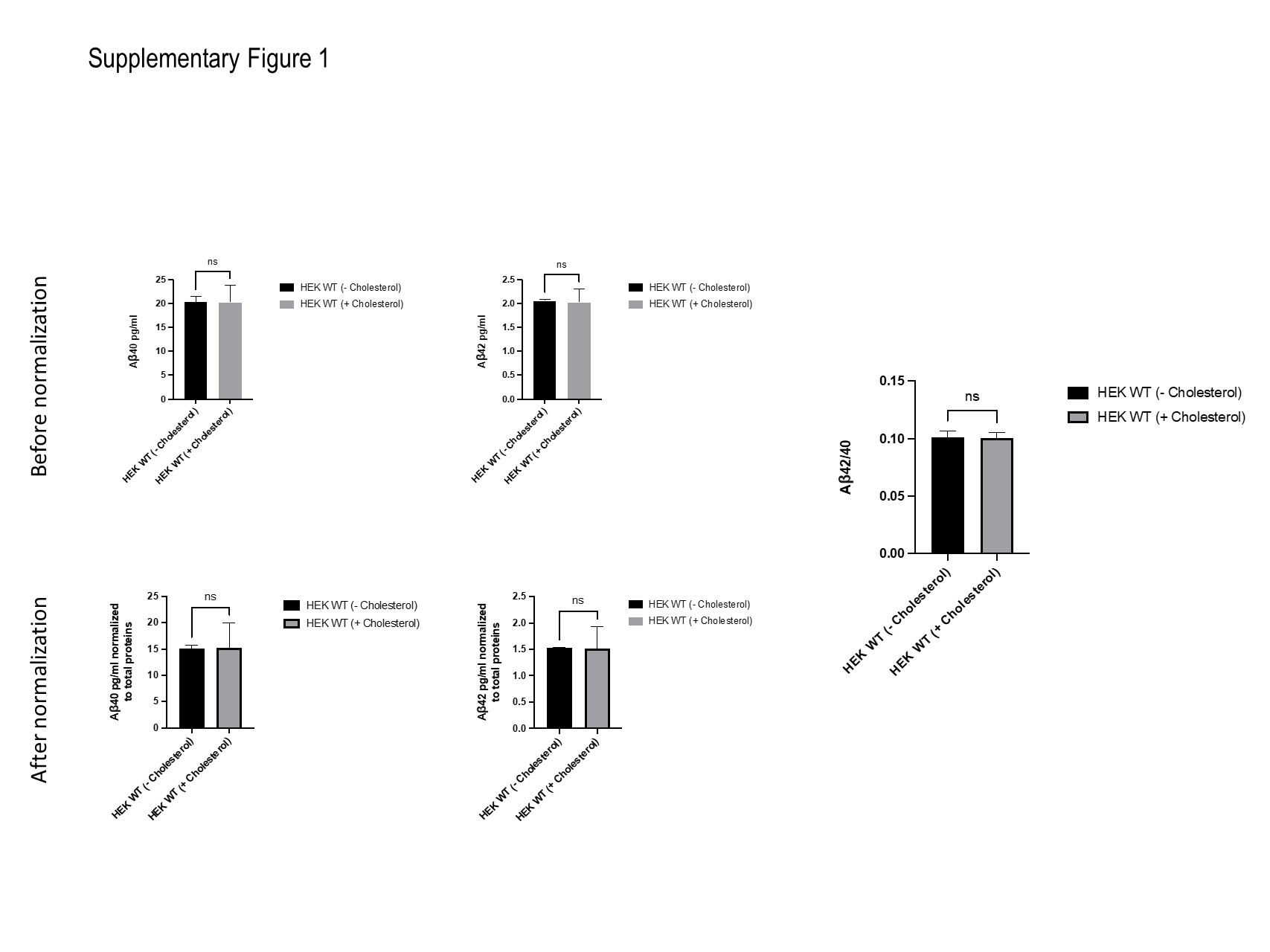

Supplement: Supplementary file 1 — Supplementary Fig. 1. Levels of Aβ40 and Aβ42 from naïve HEK293T cells in the absence and in the presence of additional methyl-β-cyclodextrin (MBCD) loaded with cholesterol. The results are normalized with the amount of intracellular proteins determined by Bradford assay and represented as a percentage of Aβ produced by HEK293T cells in the absence of MBCD loaded with cholesterol. (Unpaired t test, two tailed; 2 independent experiments/culture with 2 <n <6). (JPG 124 KB) [file 12035_2022_3025_MOESM1_ESM.jpg]

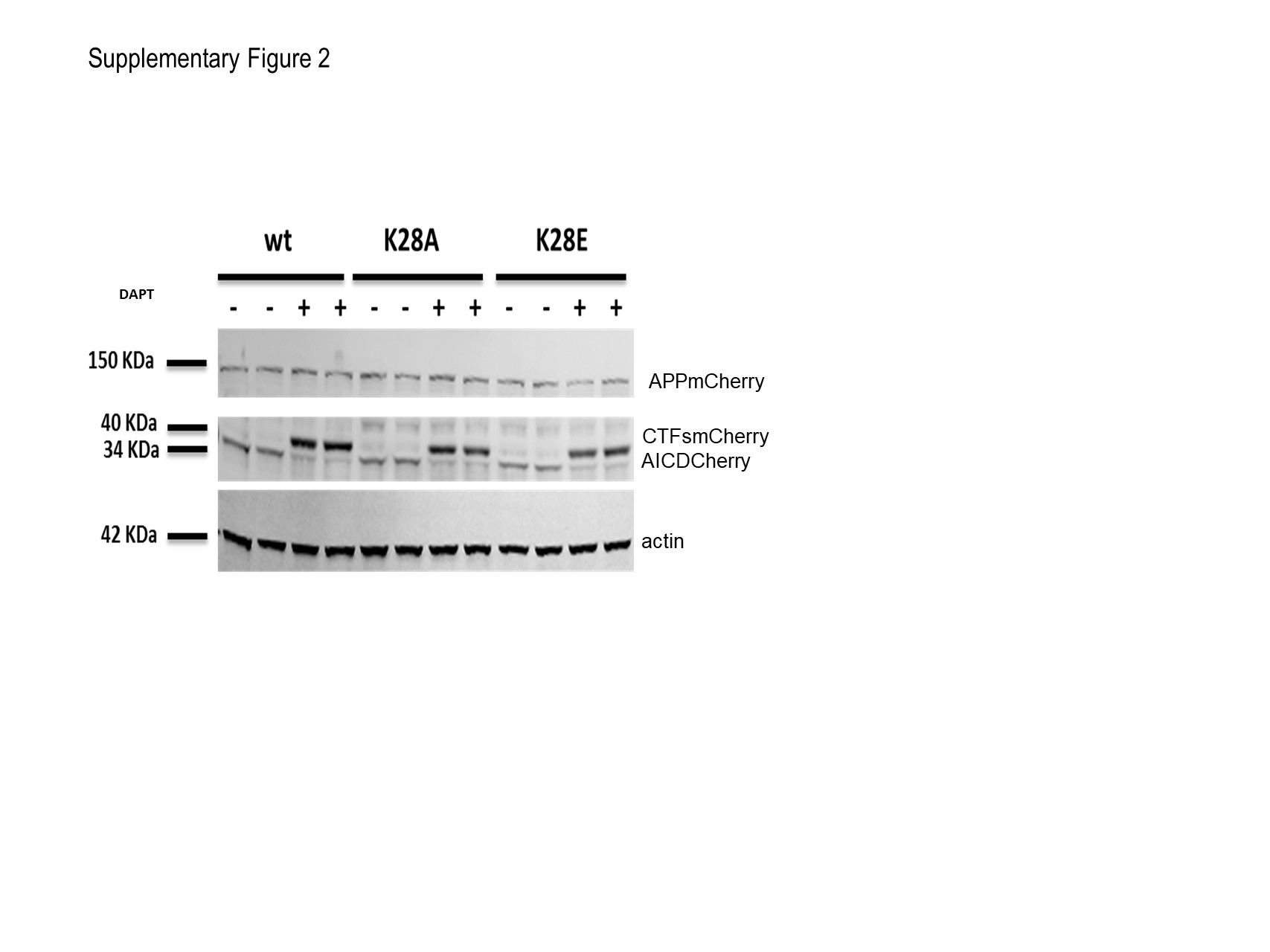

Supplement: Supplementary file 2 — Supplementary Fig. 2. Representative western blots of HEK293T cell lysates transiently transfected with APPwtmCherry, APPK28AmCherry and APPK28EmCherry mutants treated or not with the γ-secretase inhibitor DAPT hybridized with anti-APPmCherry and actin antibodies. Quantifications are in Fig. 3. (JPG 88 KB) [file 12035_2022_3025_MOESM2_ESM.jpg]

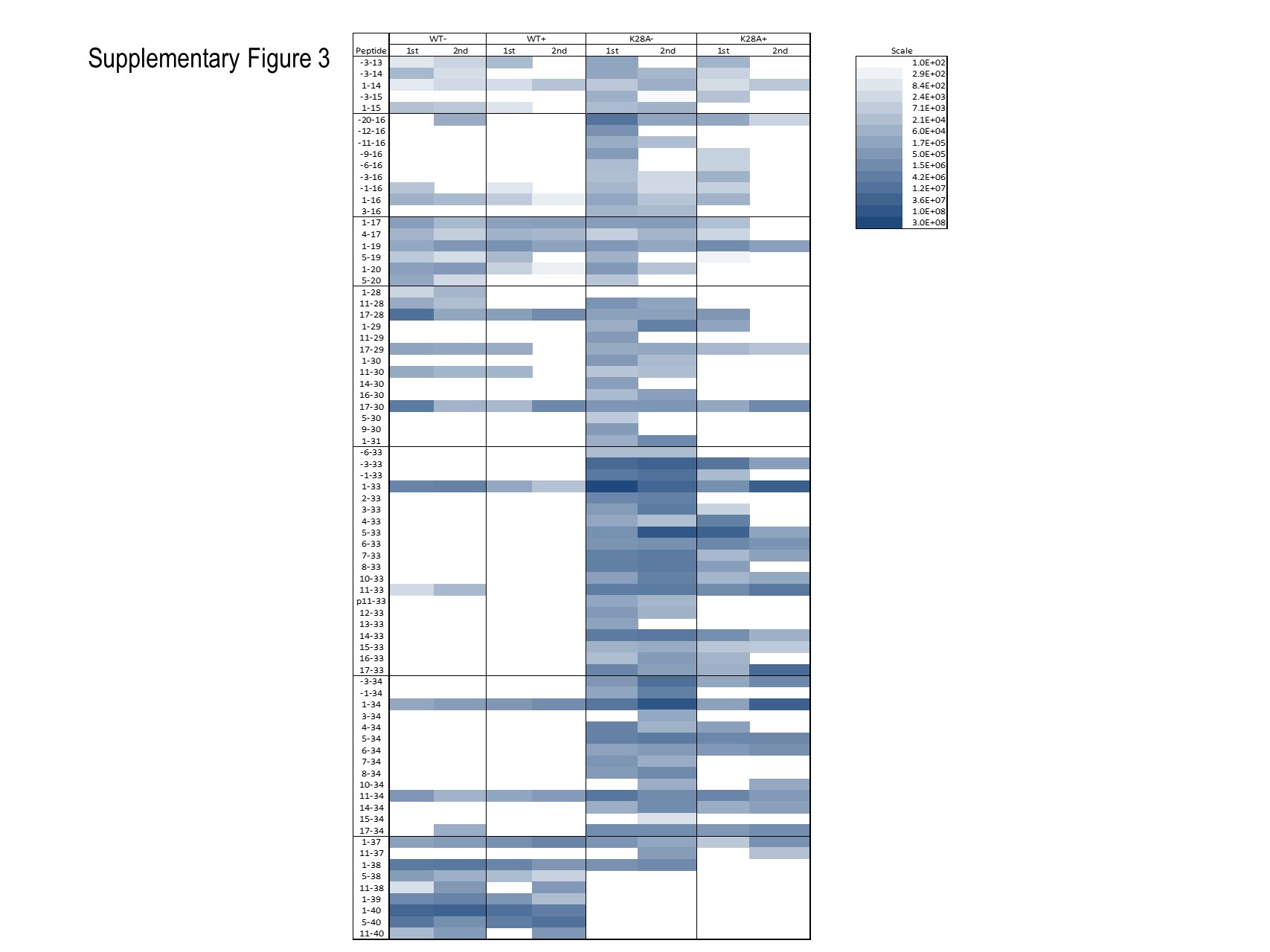

Supplement: Supplementary file 3 — Supplementary Fig. 3. Heatmap showing APP/Aβ peptides detected using LC-ESI-MS for APPWT and APPK28A treated (+) and untreated (-) with cholesterol. Peptide numbering refers to the Aβ sequence, where negative numbers indicate the number of positions N-terminally of the BACE1 cleavage site. The instensity (logarithmic scale) is the peak area normalised to total protein content in the respective sample. (JPG 166 KB) [file 12035_2022_3025_MOESM3_ESM.jpg]

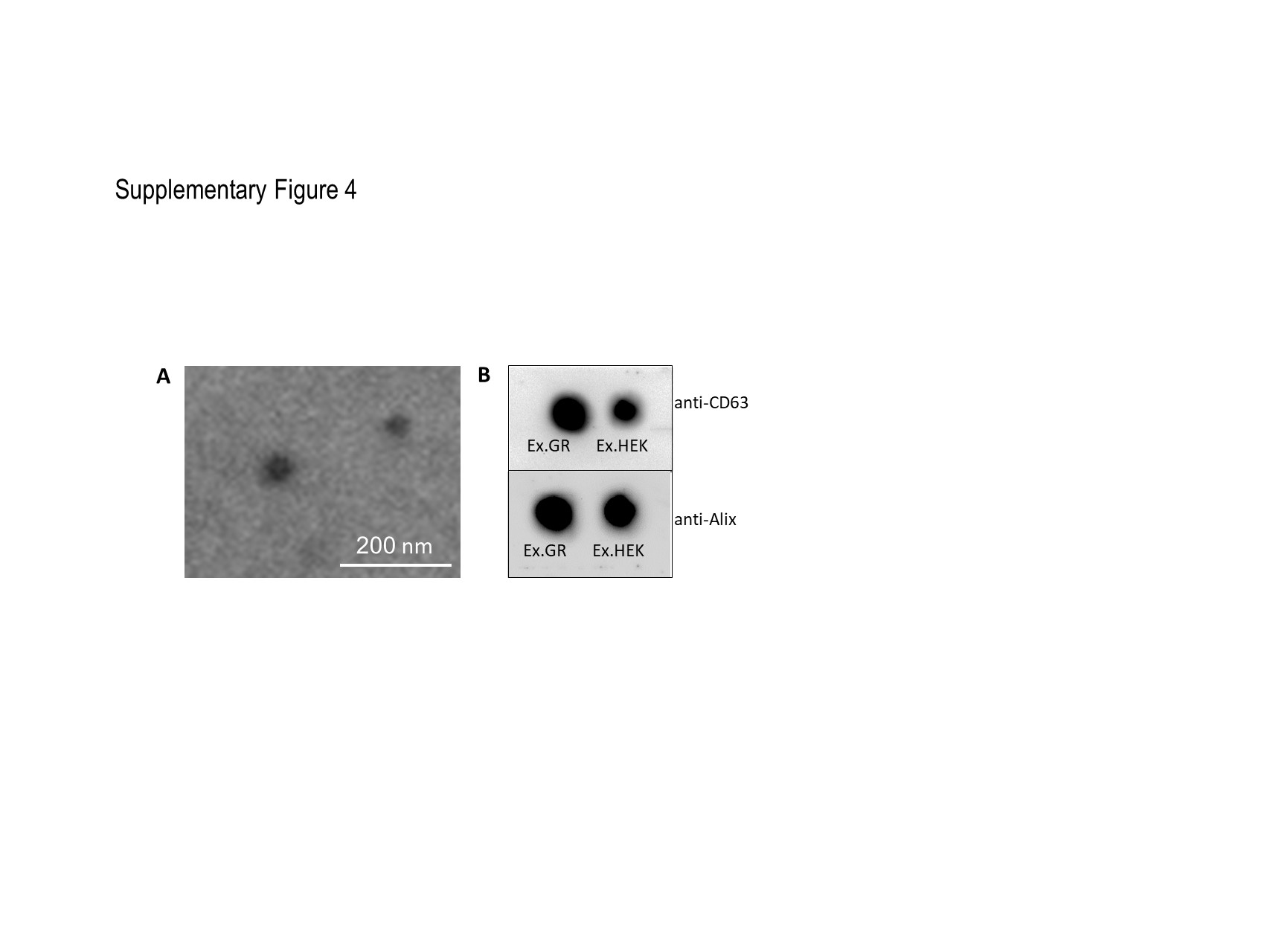

Supplement: Supplementary file 4 — Supplementary Fig. 4. Characterization of exosomes isolated from HEK293T cells. A: Transmission electron microscopy (TEM) representative image; B: Dotblot of red blood cells exosomes (Ex.GR) and of HEK293T exosomes (Ex.HEK) stained with anti-CD63 and anti-Alix antibodies (1/1000 dilution). (JPG 70 KB) [file 12035_2022_3025_MOESM4_ESM.jpg]
